# Removing batch effects from purified plasma cell gene expression microarrays with modified ComBat

**DOI:** 10.1186/s12859-015-0478-3

**Published:** 2015-02-25

**Authors:** Caleb K Stein, Pingping Qu, Joshua Epstein, Amy Buros, Adam Rosenthal, John Crowley, Gareth Morgan, Bart Barlogie

**Affiliations:** 1 Myeloma Institute for Research and Therapy, University of Arkansas for Medical Sciences, Little Rock, AR USA; 2 Cancer Research and Biostatistics, Seattle, WA USA

**Keywords:** Microarray analysis, Gene expression profiling (GEP), Batch effect, Meta-analysis, Multiple myeloma (MM), ComBat, M-ComBat

## Abstract

**Background:**

Gene expression profiling (GEP) via microarray analysis is a widely used tool for assessing risk and other patient diagnostics in clinical settings. However, non-biological factors such as systematic changes in sample preparation, differences in scanners, and other potential batch effects are often unavoidable in long-term studies and meta-analysis. In order to reduce the impact of batch effects on microarray data, Johnson, Rabinovic, and Li developed ComBat for use when combining batches of gene expression microarray data.

We propose a modification to ComBat that centers data to the location and scale of a pre-determined, ‘gold-standard’ batch. This modified ComBat (M-Combat) is designed specifically in the context of meta-analysis and batch effect adjustment for use with predictive models that are validated and fixed on historical data from a ‘gold-standard’ batch.

**Results:**

We combined data from MIRT across two batches (‘Old’ and ‘New’ Kit sample preparation) as well as external data sets from the HOVON-65/GMMG-HD4 and MRC-IX trials into a combined set, first without transformation and then with both ComBat and M-ComBat transformations. Fixed and validated gene risk signatures developed at MIRT on the Old Kit standard (GEP5, GEP70, and GEP80 risk scores) were compared across these combined data sets.

Both ComBat and M-ComBat eliminated all of the differences among probes caused by systematic batch effects (over 98*%* of all untransformed probes were significantly different by ANOVA with 0.01 q-value threshold reduced to zero significant probes with ComBat and M-ComBat). The agreement in mean and distribution of risk scores, as well as the proportion of high-risk subjects identified, coincided with the ‘gold-standard’ batch more with M-ComBat than with ComBat. The performance of risk scores improved overall using either ComBat or M-Combat; however, using M-ComBat and the original, optimal risk cutoffs allowed for greater ability in our study to identify smaller cohorts of high-risk subjects.

**Conclusion:**

M-ComBat is a practical modification to an accepted method that offers greater power to control the location and scale of batch-effect adjusted data. M-ComBat allows for historical models to function as intended on future samples despite known, often unavoidable systematic changes to gene expression data.

## Background

Multiple myeloma (MM) is a systemic hematopoietic malignancy of plasma cells that expands within the bone marrow. As plasma cells become cancerous and multiply, levels of monoclonal immunoglobulins in blood increase and osteolytic bone lesions form in the majority of patients. Myeloma is also characterized by genetic heterogeneity at onset as well as variability in patient response to treatment [1]–survival in patients ranges from months to more than fifteen years.

Gene expression profiling (GEP) via microarray analysis measures the expression levels of tens of thousands of genes simultaneously, and has been widely used in clinical practice for cancer classification, risk stratification, and treatment selection. This technique involves analyzing RNA extracted from purified plasma cells, pulled from the bone marrow, on high-density microarray gene chips [2,3]. Many gene signatures have been identified using GEP data to predict outcome of patients with newly diagnosed disease and identify those with high-risk disease. The GEP5 [4], GEP70 [5], GEP80 [6], EMC-92 [7], MRC-IX-6 [8], Millenium-100 [9], and IFM-15 [10] are all gene signatures for identifying high-risk myeloma in newly diagnosed patients. The GEP70 risk score identified 70 genes linked to shorter overall survival, duration of complete response, event free survival, and progression to clinical disease [5,11-13]. The GEP5 has recently been identified as the optimal 5-gene subset within the GEP70 [4], and the GEP80 was developed based on differentially expressed genes between baseline and 48-hours after receiving Bortezomib [6]. All three of these signatures (GEP5, GEP70, and GEP80) were trained and validated using samples from the Myeloma Institute for Research and Therapy (MIRT). Because these GEP-based risk models are affected by the expression of individual genes, systematic differences in expression due to batch effects must be corrected when comparing gene signatures across different batches.

Batch effect corrections aim to eliminate systematic, non-biological differences that may be introduced from a variety of sources. Some of the main contributors to batch effects include:

- Ambient conditions during sample preparation and handling such as room temperature, humidity, and ozone levels
- Sites/laboratories: different laboratories may have different operating procedures
- Storage/shipment conditions
- Sample preparation (including RNA isolation, amplification, and hybridization): different reagent lots may perform differently
- Scanner: type, settings, and calibration drift [14,15].

Despite knowing the many potential sources of batch effects, batch effect associated information is not necessarily recorded for all samples. Oftentimes, data analysts are left with surrogates such as processing date and preparation group to use when correcting for batch effects [16]. Here, we will generalize the term ‘batch effect’ to mean any type of systematic bias between two or more groups of samples due to differences in sample preparation, scanner, laboratory of analysis, etc. Many batch effects are unavoidable due to the large sample size requirements and potentially lengthy time required to complete a study. Combining data from different batches without correcting for batch effects can lead to, at a minimum, increased noise and less power to detect a real biological signal, and at a maximum, false biological conclusions. Careful consideration is needed in identifying and removing batch effects before further downstream analyses occur.

Combining batches of samples can be performed both within a single study or on samples from different studies in a meta-analysis framework. The remainder of this paper will focus on the latter scenario of combining larger cohorts of patients across different studies with a modified alternative of ComBat. The method introduced below, M-ComBat, would work in smaller sample, single cohort situations; however, ComBat or other methods may be more applicable in the single cohort context depending on the focus of the study.

## Methods

### Baseline MM purified plasma cell samples

All MIRT samples are baseline purified plasma cells from the bone marrow of patients enrolled on UARK Total Therapies and processed on Affymetrix U133Plus 2.0 microarrays (Santa Clara, CA) between 2004 and 2014. In keeping with institutional, federal, and Helsinki Declaration guidelines, all identifiable patients gave written informed consent for undergoing bone marrow sampling for gene expression profiling and the institutional review board of the University of Arkansas for Medical Sciences approved the research studies. Samples analyzed at the Myeloma Institute were prepared with either the One-Cycle and Two-Cycle Target Labeling and Control Reagents (‘Old’ Kit) or, beginning in 2009, the 3’ IVT Express Kit (‘New’ Kit) [17]. All baseline MM GEP MIRT data are current as of November 11, 2014. There are a total of 1071 baseline MM GEP samples included in this study from two main batches: ‘Old’ Kit samples processed at Myeloma Institute (*n*=928) and ‘New’ Kit samples (*n*=143). Additional external data sets will be used to validate both the M-ComBat method and the GEP5, GEP70, and GEP80 risk signatures. The publicly available data set of previously untreated patients (HOVON-65/GMMG-HD4, *n*=288, accession number: GSE19784, hereon referred to as HOVON-65) will be used as well as baseline data from the MRC-IX trial (*n*=273) [8,18]. Raw intensity values were MAS5 normalized and converted to log2 scale.

Here we will treat the MIRT: Old Kit data as our ‘gold-standard’, reference batch because it was used to train and validate the GEP5, GEP70, and GEP80. When using M-ComBat, the designation of the reference batch should relate to focus of investigation when combining data across batches. Here we are investigating three risk signatures (GEP5, GEP70, and GEP80) which are based on a particular standard (MIRT: Old Kit). Alternatively if we were investigating the EMC-92 risk signature, we would choose the HOVON-65/GMMG-HD4 data as the reference batch as it is the training set of the EMC-92. In a meta-analysis context where we are performing new analyses or searching for new signatures on combined data, there are no set rules on choosing a reference batch; however, it may be best to choose the largest cohort, most relevant, or most familiar as the reference batch. However, the only benefit of M-ComBat over Combat in that context is that a newly discovered signature would be fixed to a known standard rather than the arbitrary location of ComBat transformed data.

### ComBat

ComBat [19] is a highly effective method of removing batch effects based on an empirical Bayes framework that allows for borrowed strength across probes. ComBat has proven itself to be a strong method, particularly with smaller sample sizes [20], and continues to be a widely used technique [14,15,21]. Chen et al. [20] found ComBat to be ‘best able to reduce and remove batch effects while increasing precision and accuracy’ when compared against five other popular batch effect removal tools.

ComBat combines expression data by first standardizing the data, given by

$$Z_{ijg}=\frac{Y_{ijg}-\hat{\alpha}_{g}-\mathrm{X}\hat{\beta}_{g}}{\hat{\sigma}_{g}}, $$

where ordinary least-squares is used to calculate gene-wise mean and standard deviation estimates, $\hat {\alpha }_{g}$ and $\hat {\sigma }_{g}$, across gene *g*, sample *j*, and batch *i*. *Y*_*ijg*_ refers to the raw expression data, and $\mathrm {X}\hat {\beta }_{g}$ represents potential non-batch related covariates and coefficients in the model. The standardized data is assumed to be normally distributed, $Z_{\textit {ijg}}\sim N\left (\gamma _{\textit {ig}},\delta _{\textit {ig}}^{2}\right)$, where *γ*_*ig*_ and $\delta _{\textit {ig}}^{2}$ are the batch effect parameters with Normal and Inverse Gamma prior distributions, respectively. Method of moments is used to estimate hyperparameters which are used to compute the Empirical Bayes estimates of conditional posterior means gene-wise by batch for the batch effects parameters. The final batch effect adjusted data is given by

$$Y_{ijg}^{*}=\frac{\hat{\sigma}_{g}}{\hat{\delta}_{ig}^{*}}\left(Z_{ijg}-\hat{\gamma}_{ig}^{*}\right)+\hat{\alpha}_{g}+\mathrm{X}\hat{\beta}_{g}. $$

ComBat centers data to the overall, grand mean of all samples which results in an adjusted data matrix that is shifted to an arbitrary location that no longer coincides with the location of any original batch. When using validated and fixed gene signatures on ComBat transformed data, these gene signatures on ‘gold-standard’ data will also be shifted. Many of these ‘gold-standard’ samples were likely used to train and build the gene signature; therefore, altering these samples when combining data sets will also alter the performance and proportion of high-risk individuals identified for a fixed risk signature on the original, reference training data.

### M-ComBat

In this study, we propose a modified version of ComBat (M-ComBat) that shifts samples to the mean and variance of the ‘gold-standard’, reference batch, rather than the grand mean and pooled variance. This is achieved by changing the standardizing mean and variance estimates, $\hat {\alpha }_{g}$ and $\hat {\sigma }_{g}$, to batch-wise estimates, $\hat {\alpha }_{\textit {ig}}$ and $\hat {\sigma }_{\textit {ig}}$, such that the standardized data is then given by

$$Z_{ijg}=\frac{Y_{ijg}-\hat{\alpha}_{ig}-\mathrm{X}\hat{\beta}_{g}}{\hat{\sigma}_{ig}}. $$

Furthermore, the mean and variance estimates used in the final batch effect adjusted data are calculated using the gene-wise mean and variance estimates of the ‘gold-standard’, reference batch, *i*=*r*.

The M-ComBat adjusted data is then given by

$$Y_{ijg}^{*}=\frac{\hat{\sigma}_{i=r,g}}{\hat{\delta}_{ig}^{*}}\left(Z_{ijg}-\hat{\gamma}_{ig}^{*}\right)+\hat{\alpha}_{i=r,g}+\mathrm{X}\hat{\beta}_{g}. $$

M-ComBat can be applied in *R* using a modified version of the ComBat script from the *sva* package. The altered script (including a small, simulated example) is available online for public use at http://github.com/SteinCK/M-ComBat.

We will illustrate both ComBat and M-ComBat as well as the GEP5, GEP70, and GEP80 risk signatures by transforming baseline purified plasma cell GEP samples from UARK Total Therapies across two kinds of sample preparation (Old and New kits) as well as two external data sets (HOVON-65 and MRC-IX). ComBat will be performed assuming a parametric model with no covariates. The four distinct batches will be shifted by M-ComBat to the MIRT: Old Kit ’gold-standard’ as this was the standard of data used to develop and train the GEP5, GEP70, and GEP80 signatures.

## Results

Both ComBat and M-ComBat completely eliminated significant batch effect related differences across the four distinct batches. Prior to transformation, over 98*%* of all probes were significantly differently expressed across at least one batch (probe-wise ANOVA found 53,827 of 54,675 q-values [22] below a 0.01 false discovery rate threshold). After performing either ComBat or M-ComBat, zero probes remained significantly differently expressed across batches according to the same threshold.

**Figure 1:**
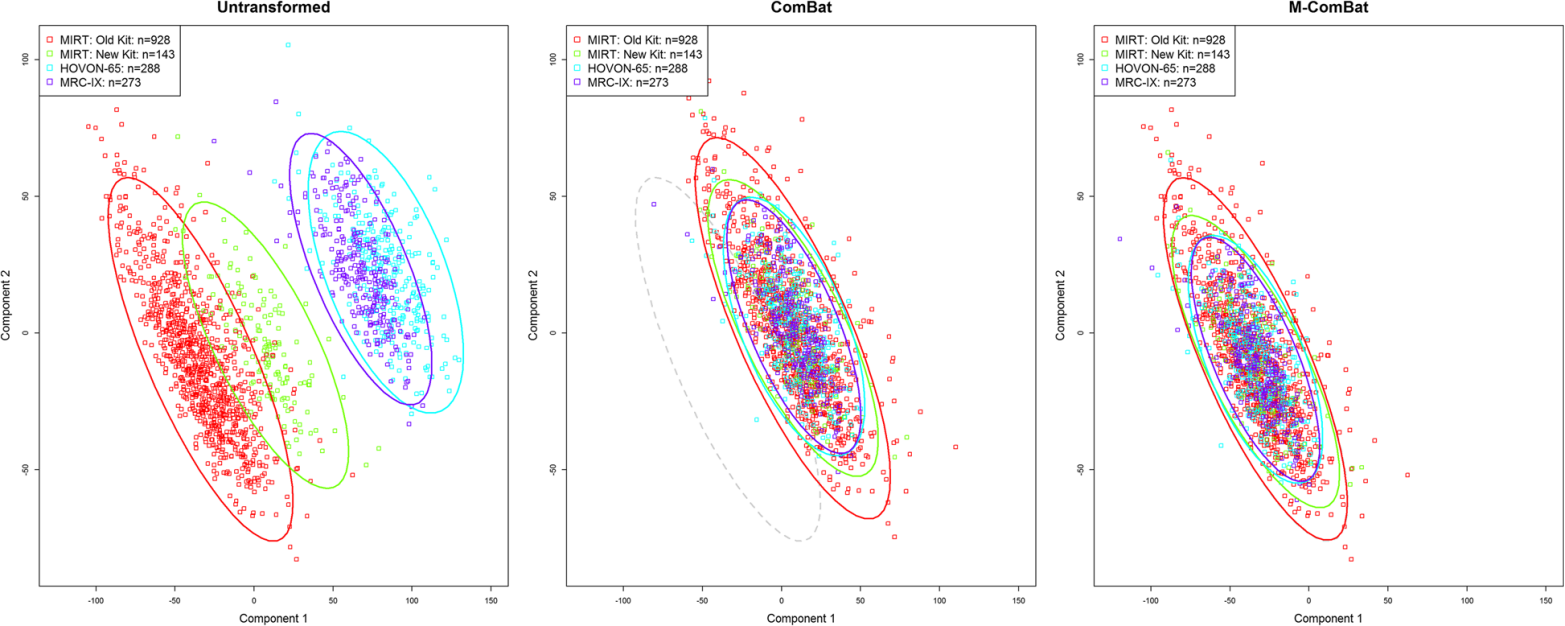
**Principal component analysis.** Scatter plot of Principal Component Analysis (PCA) of the 5,000 most variable probes across batches without transformation, transformed by ComBat, and transformed to the MIRT: Old Kit standard by M-ComBat. Grey data ellipse seen in ComBat panel represents untransformed MIRT: Old Kit. After transformation by M-ComBat, the differences in location between the batches has been eliminated and all batches coincide with the desired MIRT: Old Kit standard.

In order to further investigate the differences between ComBat and M-ComBat transformation, principal component analysis (PCA) was performed on the 5,000 most variable probes for the untransformed, ComBat, and M-ComBat transformed data sets. PCA creates convex linear combinations of a set of observations that are orthogonal and defined in such a way that the components are ordered by variance where the first principal component has the largest variance, the second the next largest variance, etc. Scatterplots of the top two principal components show how both ComBat and M-ComBat remove the differences in probe expression between the batches while shifting the data to different locations (Figure 1). ComBat transformed PCA plot includes a grey ellipse marking the ‘gold-standard’ 95*%* data ellipse of the untransformed MIRT: Old Kit data. By shifting all of the data to the grand mean rather than a specific batch mean, ComBat alters the location of potential reference samples and therefore also alters gene signatures developed on those reference samples. However following transformation by M-ComBat, all three batches overlay one another and are centered on the MIRT: ‘Old’ Kit batch. This visually demonstrates the two main features of M-ComBat: elimination of batch effect related differences while offering researchers the ability to shift data to a desired standard.

**Figure 2:**
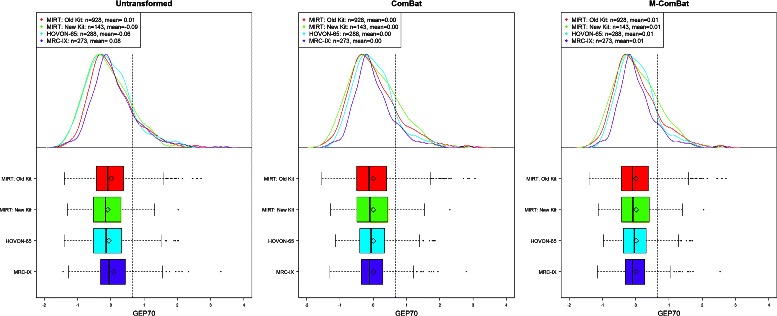
**GEP70 boxplots and density curves.** Boxplot and density curves of GEP70 scores for all baseline MM purified plasma cell samples across four batches without transformation, transformed by ComBat, and transformed to the MIRT: Old Kit standard by M-ComBat. Grey, dashed line indicates high risk threshold for GEP70. Following transformation by ComBat and M-ComBat, GEP70 scores are more aligned in mean, median, and distribution across batches.

**Figure 3:**
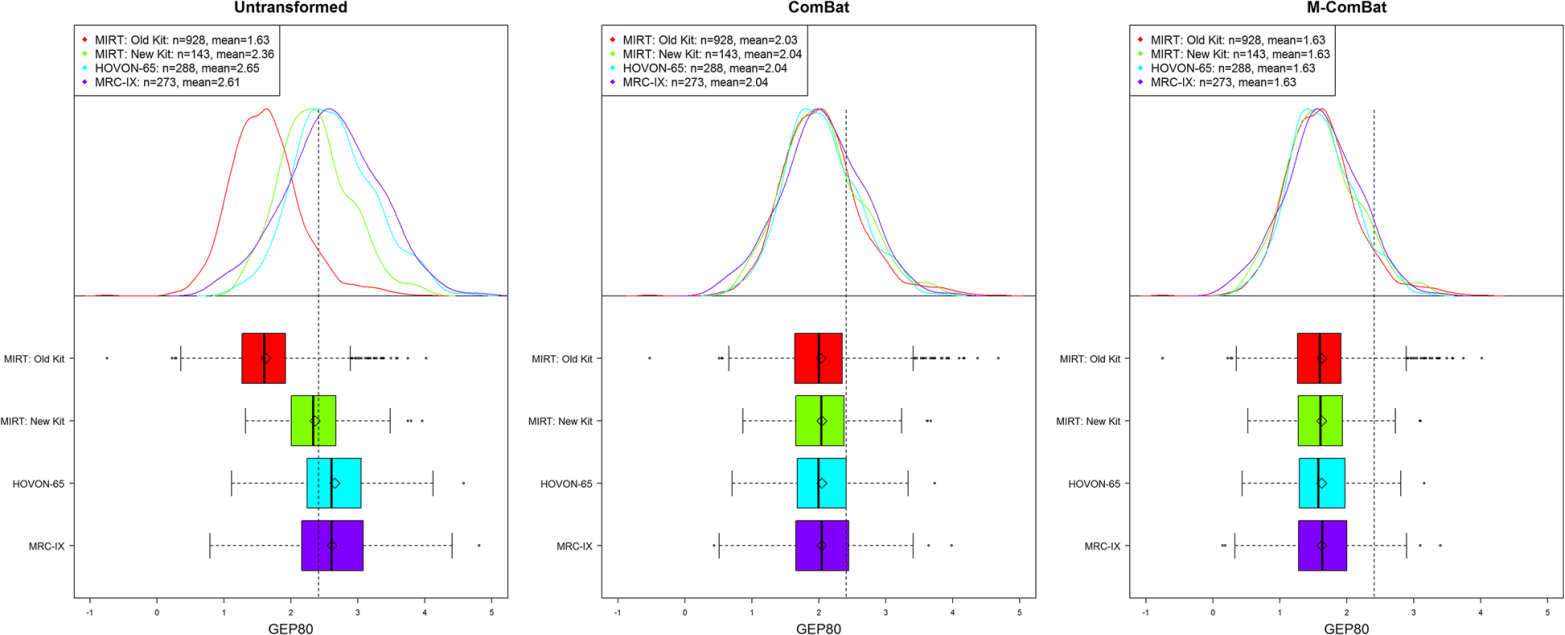
**GEP80 boxplots and density curves.** Boxplot and density curves of GEP80 scores for all baseline MM purified plasma cell samples across four batches without transformation, transformed by ComBat, and transformed to the MIRT: Old Kit standard by M-ComBat. Grey, dashed line indicates high risk threshold for GEP80. Following transformation by ComBat and M-ComBat, GEP80 scores are more aligned in mean, median, and distribution across batch.

Differences in the distribution of risk scores across batches are eliminated for GEP5, GEP70, and GEP80 using either ComBat or M-ComBat (Figures 2 and 3). M-ComBat also shifts the means of each risk score back to the mean of the original untransformed, ‘gold-standard’ batch. The mean GEP70 score for all batches after M-ComBat is 0.01, which is the same as the untransformed MIRT: Old Kit mean GEP70 score (identical effect for GEP5 and GEP80). The GEP70 also shows that it is better protected from the impact of batch effects as its distribution across batches are more aligned for the untransformed data (than GEP5 or GEP80) and change minimally following ComBat or M-ComBat.

**Figure 4:**
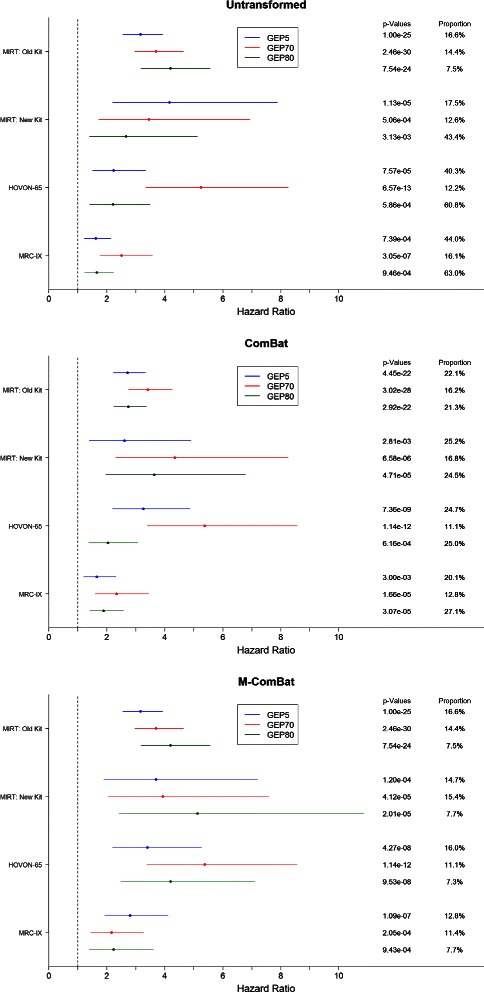
**Forest plot of cox proportional hazards models.** Forest plot of overall survival hazard ratios associated with cox proportional hazards of GEP5, GEP70, and GEP80 risk across four batches without transformation, transformed by ComBat, and transformed to the MIRT: Old Kit standard by M-ComBat. Included are the Wald p-values and proportion of high-risk individuals for each score by original risk thresholds.

The GEP80 is more susceptible to the impact of batch effects, especially in the proportion of patients designated with high risk. The proportion of high-risk GEP80 patients on the untransformed MIRT: Old Kit is 7.5*%*, while all other non-transformed data sets have over 43*%* of patients defined as high-risk (using optimal high-low risk cutoff from original research) (Figure 4). When transforming all four of these batches by ComBat, all four data sets define at least 21*%* of individuals as high risk including our ‘gold-standard’ batch; however, when using M-ComBat all four of these batches move within 7.3*%* to 7.7*%* defined as high risk. These proportions of high risk are much closer to those seen on the untransformed, ‘gold-standard’ MIRT: Old Kit data (7.5*%*). A similar trend occurs for the other scores, and in general M-ComBat allows for original optimal cutpoints to continue to function well on external data sets for binary, high-low risk scores. Alternatively, ComBat adjusts all data sets, so the proportion of high-risk individuals may change–even in our reference, ‘gold-standard’ batch.

**Figure 5:**
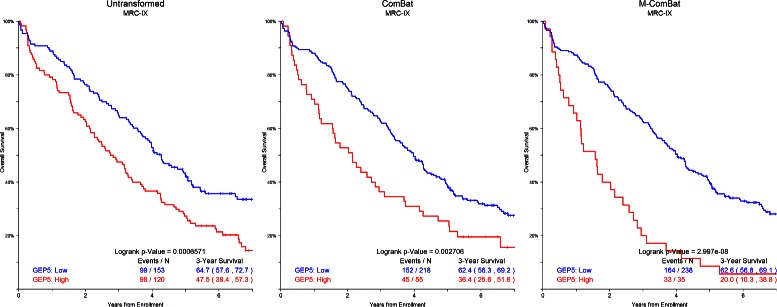
**GEP5 Kaplan-Meier curves for MRC-IX.** Kaplan-Meier plot of GEP5 risk for MRC-IX data without transformation, transformed by ComBat, and transformed to the MIRT: Old Kit standard by M-ComBat. GEP5 risk for MRC-IX data is most associated with survival when transformed to MIRT: Old Kit standard. Overall logrank p-values are included as well as 3-year survival estimates with 95*%* confidence intervals.

**Figure 6:**
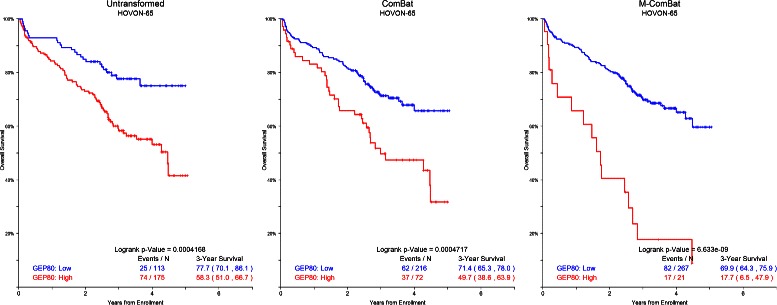
**GEP80 Kaplan-Meier curves for HOVON-65.** Kaplan-Meier plot of GEP80 risk for HOVON-65 data without transformation, transformed by ComBat, and transformed to the MIRT: Old Kit standard by M-ComBat. GEP80 risk for HOVON-65 data is most associated with survival when transformed to MIRT: Old Kit standard. Overall logrank p-values are included as well as 3-year survival estimates with 95*%* confidence intervals.

In general, hazard ratios from Cox regression of overall survival increase and Wald p-values decrease following ComBat and M-ComBat transformation (Figure 4). The GEP5 applied to MRC-IX (Figure 5) and the GEP80 on the HOVON-65 data (Figure 6) showed significant improvements between ComBat and M-ComBat transformations. In both scenarios, using M-ComBat and the original high-low cutoffs allowed for increased ability to identify individuals that are truly high risk (3-year survival from ComBat to M-ComBat decreases 16.4*%* for MRC-IX and 32.0*%* for HOVON-65). M-ComBat allows for improved performance of risk scores on external data sets and allows for risk scores to function as intended.

## Discussion

M-ComBat eliminated the overwhelming statistical differences among probes caused by systematic batch effects. In addition to eliminating differences in probes, the agreement in mean and distribution of risk scores coincided with that of the ‘gold-standard’ batch with M-ComBat. The proportion of high-risk patients identified following M-ComBat for external data sets more closely matched that of the reference group as well. The performance of risk scores improved overall using either ComBat or M-Combat; however, using M-ComBat and the original, optimal risk cutoffs allowed for greater ability in our study to identify a smaller cohort of high-risk subjects.

By eliminating the differences in means between all probes, all mean risk scores examined coincided with one another across batches following M-ComBat. This allows for potentially increased power in the risk score on external data sets following M-ComBat transformation. M-ComBat provides the structure to compare risk scores more fairly and realistically across different data sets lending itself directly to the further validation and comparison of yet discovered gene signatures scores.

## Conclusion

Modified ComBat eliminates batch effect related differences while adjusting all samples to a pre-defined standard. The modification transforms samples to the mean and variance of the ‘gold-standard’ batch without altering samples from the original batch. This allows for validated and fixed predictive models to function properly on external data sets and offers a less biased framework for comparing gene signatures. Rather than redefining cutpoints of GEP-based scores for new batches, M-ComBat allows for the same cutpoints and logic used to build these GEP-based scores to function properly on external data sets.

Differences in scanner, laboratory, and sample preparation contributed significantly toward systematic batch effects seen when combining gene expression samples across these four batches. The non-biological variation introduced by differences in sample preparation kit, scanner, and laboratory had dramatic impacts on overall GEP expression (seen in PCA plot) as well as the smaller subsets of probes that define gene signatures. These variations are often unavoidable in long-term studies, and both ComBat and M-ComBat are equally as effective in eliminating these confounding batch effect related differences. However, M-ComBat offers an intelligent framework to overcome these changes and the power to control the location and scale of the transformed data.

M-ComBat allows for gene expression meta-analyses to combine data from different studies to a known standard. The benefit of a fixed standard in meta-analysis is important for gene signature comparison, differential expression analysis, and the search for new gene signatures on a larger subset of combined samples on a known standard.

M-ComBat is a practical modification to an accepted method that offers greater power to control the appearance of batch-effect adjusted data. M-ComBat allows for historical models to function as intended on future samples despite known, often unavoidable systematic changes to gene expression data.
